# Time Efficiency of Digitally and Conventionally Produced Single-Unit Restorations

**DOI:** 10.3390/dj9060062

**Published:** 2021-06-01

**Authors:** Sofia Stromeyer, Daniel Wiedemeier, Albert Mehl, Andreas Ender

**Affiliations:** 1 Division of Computerized Restorative Dentistry, Clinic for Conservative and Preventive Dentistry, Center of Dental Medicine, University of Zurich, 8032 Zurich, Switzerland; Albert.Mehl@zzm.uzh.ch (A.M.); Andreas.Ender@zzm.uzh.ch (A.E.); 2 Statistical Services, Center of Dental Medicine, University of Zurich, 8032 Zurich, Switzerland; Daniel.Wiedemeier@zzm.uzh.ch

**Keywords:** CAD/CAM, digital workflow, conventional workflow, chairside, labside, time measurement

## Abstract

The purpose of this in vitro study was to compare the time efficiency of digital chairside and labside workflows with a conventional workflow for single-unit restorations. The time efficiency in this specific sense was defined as the time, which has to be spent in a dental office by a dental professional performing the relevant steps. A model with interchangeable teeth on position 36 was created. These teeth were differently prepared, responding to several clinical situations to perform single-unit restorations. Different manufacturing techniques were used: For the digital workflows, CEREC Omnicam (CER) and Trios 3 (TN/TI) were used. The conventional workflow, using a dual-arch tray impression technique, served as the control group. For the labside workflow (_L) and the conventional impression procedure (CO), the time necessary for the impressions and temporary restorations was recorded and served as operating time. The chairside workflow time was divided by the time for the entire workflow (_C) including scan, design, milling and finishing the milled restoration, and in the actual working time (_CW) leaving out the chairside milling of the restoration. Labside workflow time ranged from 9 min 27 s (CER_L) to 12 min 41 s (TI_L). Entire chairside time ranged from 43 min 35 s (CER_C) to 58 min 43 s (TI_C). Pure chairside working time ranged from 15 min 21 s (CER_CW) to 23 min 17 s (TI_CW). Conventional workflow time was 10 min 39 s (CO) on average. The digital labside workflow and the conventional workflow require a similar amount of time. The digital chairside workflow is more time consuming.

## 1. Introduction

The use of computer-aided systems in dentistry has increased in the last 10 years and has led to an alternative approach to the conventional workflow with respect to the intraoral impressions [1,2,3,4,5,6,7,8]. Nowadays, the 3D data of an intraoral scanner can be further processed in almost every branch of dentistry by combining with specific software [2,3,5,7,9,10]. For example, computer-aided design (CAD)/computer-aided manufacturing (CAM) allows high-quality restorations to be fabricated economically and aesthetically [1,2,4,5,10,11,12]. The digital workflow begins with the digitization of the tooth to be restored [1,2,5]. Subsequently, there are two possibilities for producing restorations using CAD/CAM, either via a dental laboratory (labside) or directly in the dental office (chairside) [2,5,13]. In the labside workflow, the dataset containing the information about the digital model is sent to a dental laboratory where the restoration is designed virtually with the aid of CAD software [1,2]. This designed restoration is then sent to a milling device that produces the dental restoration (CAM) [1,2,7]. In the chairside workflow, the whole digital workflow takes place in the dental office; the premise is an intraoral scanner with a CAD software and a milling machine on site. So the dentist can carry out every manufacturing step (scan, design and milling) [1,2,5,14]. Taking dental impressions in the conventional workflow is one of the most important procedures and is seen as the gold standard, since good results are achieved with respect to accuracy and precision in every indication [15,16,17,18,19,20,21]. Various studies have been done regarding the accuracy of the digital workflow that shows that restorations of equal fit can be produced for a wide range of indications [4,13,14,21,22,23,24]. Other studies have compared digital scanning with conventional impression-taking with respect to time efficiency [15,18,19,21,25,26], but only a few studies have examined the entire workflow [17,27,28]. With the latest developments and a broader variety of scanners, new investigations should be conducted in this field. The aim of this in vitro study was to examine the time efficiency of the labside and chairside workflow in comparison with a conventional workflow for single-unit restorations. The time efficiency in this specific sense was defined as the time, which has to be spent in a dental office by a dental professional performing the relevant steps. The null hypothesis was that the time efficiency of labside and chairside workflows does not differ from that of the conventional workflow.

## 2. Materials and Methods

This in vitro study was carried out by a dentist-graduate with moderate skills in using CAD/CAM devices as well as in taking conventional impressions. The study had a training phase of 3 days in which both skills were improved. Data acquisition was performed using an in vitro model. All details regarding the used materials are shown in Table 1 and marked in the text with a superscripted letter (e.g., material^x^).

### 2.1. Model Fabrication

**Figure 1:**
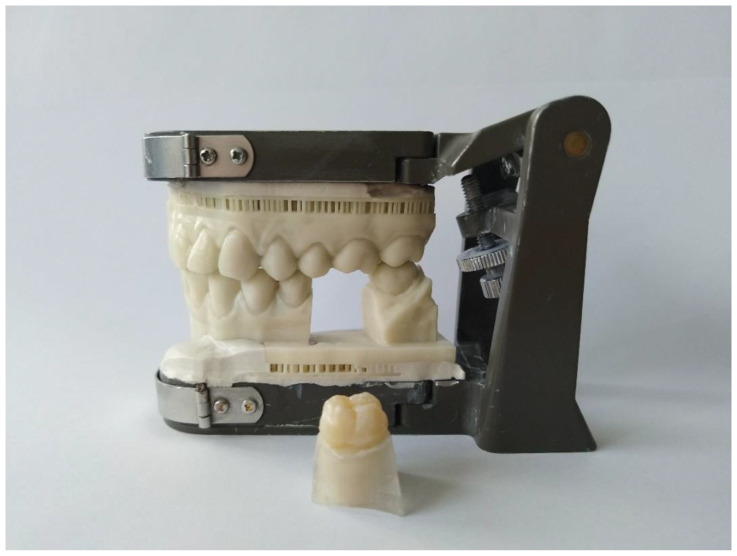
The test model with several interchangeable PMMA sockets and one of three different preparation geometries of the natural teeth (*n* = 10).

10 extracted non-decayed first lower molars were selected. These teeth were fixed^a^ in sockets^b^, which were placed in a 3D-printed model^c^ in the first lower molar position (tooth 36) (Figure 1). The extracted teeth were numbered (from 1 to 10) and then prepared using rotating diamond-coated preparation instruments. Ten different preparations were made: four inlays (mod cavity), four partial crowns (always with one or two cusps removed) and two full-crown preparations. The preparation of the teeth was conducted according to all-ceramic preparation guidelines [1,5,10,11,13,29,30].

### 2.2. Overview of the Different Workflows

**Figure 2:**
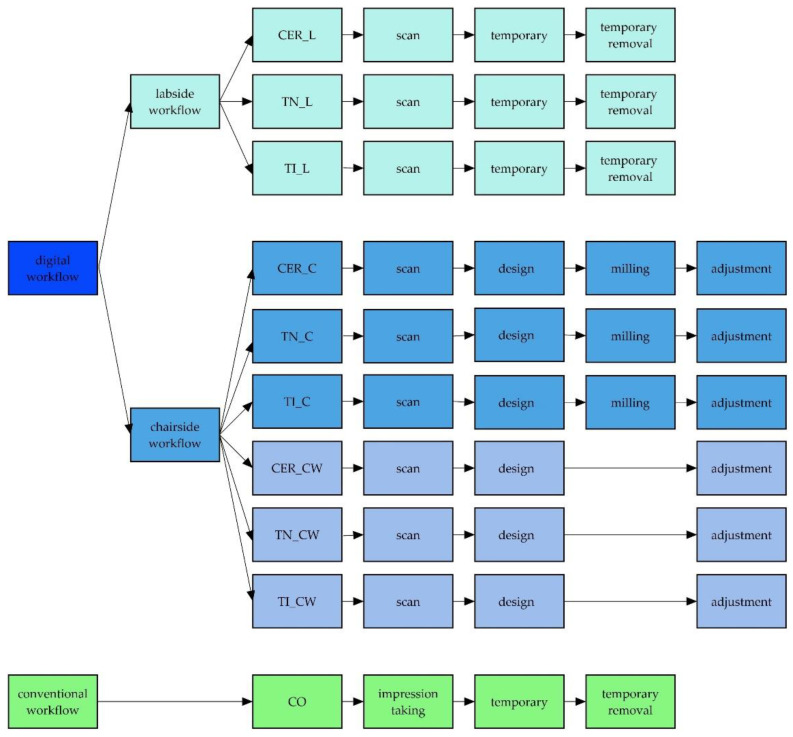
Workflows and test groups with their individual processing steps.

Two intraoral scanners were used for the labside (_L) and chairside workflow (_C/_CW): CEREC Omnicam^d^ (CER) and the Trios 3^e^ with two modes of operation (Trios normal mode (TN)/Trios insane mode (TI)). For the chairside workflow, the data of the intraoral scanners were further processed with the respective CAD/CAM software^f,g^ and the respective milling machine^h,i^. The chairside workflow time was divided in the time for the entire workflow (_C) including scan, design, milling, and finishing the milled restoration and in the actual working time (_CW), leaving out the chairside milling of the restoration. The conventional workflow (CO), using a dual-arch tray, served as the control group. Cementation of the finished restoration was not carried out since it is the same working step for every workflow. The time for every step performed during the workflow according to Table 2 and Figure 2 was measured using a stop clock.

### 2.3. Labside Workflow

For the digital scan, the lower jaw with the preparation was scanned first followed by the opposing jaw and then the buccal bite registration was made. The recommended scanning strategy was followed for each system. The Trios 3 scanning software^g^ includes two acquisition modes, the normal mode (TN) and the insane mode (TI). In the TN groups (normal scan mode), the preparation site was scanned using the high-resolution feature “HiRes” with an increased scan depth. The insane mode (TI) is for fast scanning and did not have the feature of “HiRes”. The temporary inlays and partial crowns were made of a light-curing one-component material^j,k^. Prefabricated temporary composite crowns^l^ with a temporary cement^m^ were used for the crown dies. All temporary restorations were checked for preliminary contacts during occlusion and, if necessary, were adjusted. The temporary restorations were removed and the cavity or die was cleaned. The time taken to fabricate, insert, adjust, and remove the temporary restoration was carried out one time for all sample teeth (from 1 to 10).

### 2.4. Chairside Workflow

For the labside and chairside workflow, the same scans were used. However, in the chairside workflow, these scans were processed further. In the CAD design, the model was articulated, then the preparation margin was drawn and the insertion axis determined. After the design process, the restoration was positioned inside the ceramic block for the subsequent milling process (CAM). The sequence used to edit the digital model was very similar both for CER_C and TN_C/TI_C. The major difference was that the restoration proposal in CER_C was based on the biogeneric occlusal surface design [5,13,31,32]. In TN_C/TI_C, an additional menu item was selected, in which different tooth shapes were available (tooth database), if the proposed shape was not suitable. Occlusal contacts were reduced to 0 µm and the approximal contacts to 50 µm penetration. During the CAM preparation process, the sprue in CER_C was moved horizontally to find a position where the contact point of the restoration was not compromised. Then, the extra-fine milling mode was selected. The sprue in TN_C/TI_C could be moved in the vertical and horizontal directions to find a position where the contact point of the restoration was not compromised. Then, the very high milling mode was selected.

Each restoration was milled once in the test groups CER_C and TI_C. For the group TN_C, the milling times were considered equal to TI_C, because the same milling machine would have been used. The above mentioned milling machines^h,i^ were used to produce restorations with monochromatic Feldspar-blank Vita Mark ll I14^n^.

After the restoration had been milled^o,p^, the sprue was removed and the restoration was tried in. If the restoration did not fit, the approximal contacts were adjusted. The use of dental floss was needed to ensure that there was enough contact with the neighboring teeth. The occlusion was checked. Sometimes grinding was necessary to achieve equal occlusal contacts at the restoration and neighboring teeth. Finally, the restoration was polished with two types of ceramic polishing coups^q^ and polishing paste^r^. The adjustment was only carried out in the chairside workflow because in the labside and conventional workflow this step is executed almost entirely by the dental technician. 

### 2.5. Chairside Workflow Working Time

The whole chairside workflow working time was calculated and was not carried out manually. Therefore, -CW groups exhibit equal time to _C groups without the milling time of the respective milling unit.

### 2.6. Conventional Workflow

In the conventional workflow (CO), a triple tray^s^ was chosen for the experimental model. A universal adhesive^t^ was applied to this tray and the preparation was coated with a low viscosity vinylsiloxanether^u^. The tray was filled before with a high viscosity vinylsiloxanether^v^ and then placed on the model. The setting time was not measured because the temperature in the oral cavity is usually higher than the ambient temperature, which leads to faster setting of the impression material [21]. Hence, the time specified by the manufacturer was used instead. Following setting, the tray was removed. The time for the fabrication of temporary restorations was considered the same in the labside and the conventional workflow. So this working step was carried out just one time for all sample teeth.

### 2.7. Statistics

The time recorded for each working step and for each group was documented in an Excel table and processed using the statistical software R and the PMCMR package [33,34]. Friedman tests (α < 0.05) were performed to investigate potential differences between the systems in each work-flow. If significant differences were detected, post hoc Conover tests were performed for pairwise comparison of the systems. Resulting p-values were adjusted according to Holm.

## 3. Results

**Figure 3:**
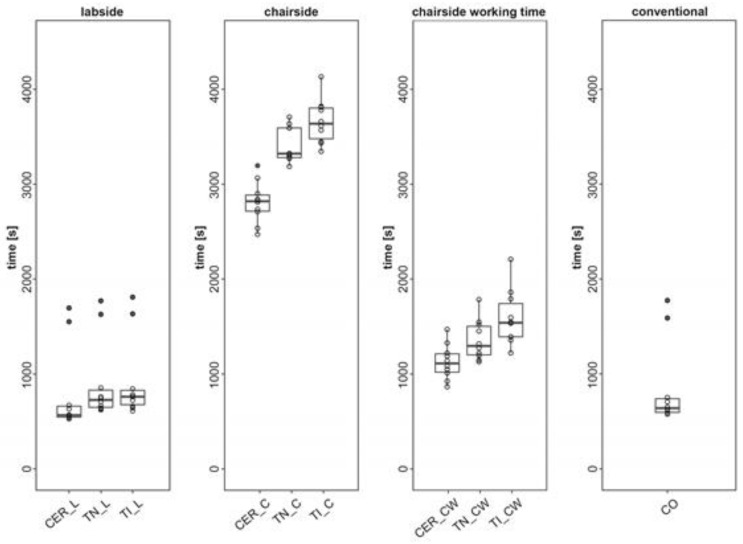
Boxplot of the workflows and their test groups.

The times recorded for all test groups, their median, and interquartile ranges (IQR) are shown in Table 3. Figure 3 is a boxplot that visually shows the differences in times between the groups.

### 3.1. Labside Workflow

In the labside workflow, all the groups differed significantly from each other (*p* < 0.05). The times measured: CER_L = 9 min 27 s (median = 09 min 27 s/IQR = 1 min 54 s); TN_L = 12 min 07 s (median = 12 min 7 s/IQR = 3 min 2 s); and TI_L = 12 min 41 s (median = 12 min 41 s/IQR = 2 min 31 s)

### 3.2. Chairside Workflow

In the chairside workflow, all CAD/CAM groups differed significantly from each other (*p* < 0.05). The times measured were: CER_C = 47 min 0 s (median = 47 min 0 s/IQR = 2 min 50 s); TN_C = 55 min 22 s (median = 55 min 22 s/IQR = 5 min 12 s); and TI_C = 60 min 38 s (median = 60 min 38 s/IQR = 5 min 24 s).

### 3.3. Chairside Workflow Working Time

Considering only the working time in the chairside workflow, all the CAD/CAM groups differed significantly from each other (*p* < 0.05). The times measured were: CER_CW = 18 min 32 s (median = 18 min 32 s/IQR = 3 min 13 s); TN_CW = 21 min 36 s (median = 21 min 36 s/IQR = 5 min 1 s); and TI_CW = 25 min 40 s (median = 25 min 40 s/IQR = 5 min 51 s).

### 3.4. Conventional Workflow

The duration of the conventional workflow differed significantly from all labside and chairside workflows except CER_CW (*p* = 0.288). The time measured for CO was 10 min 39 s (median = 10 min 39 s/IQR = 2 min 24 s)

## 4. Discussion

The aim of this in vitro study was to examine the time efficiency of the labside workflow and chairside workflow compared with a “fast track” conventional workflow for single-unit restorations. The time efficiency in this specific sense was defined as the time, which has to be spent in a dental office by a dental professional performing the relevant steps. The null hypothesis that the labside and chairside workflow do not differ in time efficiency compared to the conventional workflow was rejected.

The results of the present in vitro study should be interpreted according to their clinical relevance rather than their statistical significance due to the relatively small number of sample teeth and because of the data distribution in the nonparametric analysis. The duration of the labside and conventional workflow was similar (9 min 27 s–12 min 41 s versus 10 min 39 s). In contrast, the chairside workflow was significantly slower than the conventional one, with CO (10 min 39 s) being faster than all chairside groups (47–60 min). The milling process in the chairside workflow is the most time-consuming factor, as demonstrated in the current study and in the studies of Gozdowski [27] and Wurbs [28]. When considering only the working steps involving a dentist’s interaction (chairside working time) within the chairside workflow, there was a major reduction in time ex-penditure (18 min 32 s–25 min 40 s). Regarding the labside workflow, there have been many studies with disparate opinions about the time effectiveness of digital impressions. Benic [18] and Wismeijer [25] found that conventional impression taking was faster than digital impression taking for the fabrication of lithium disilicate single crowns and supra-structures of implants. However, Patzelt [15], Lee & Gallucci [17], Ahrberg [19] and Joda [21,26] found that digital impression taking was faster than the conventional method for single abutments, single-implant restorations, and single crowns. These differences may reflect the use of different impression trays in the conventional workflow [8,15,18,19,20,21,26]. In our study, a triple tray technique was used, which can be considered as a fast impression technique because the preparation site, the neighboring teeth, and the opposing arch can be captured in one single impression [14]. Operated in the posterior regions of the dental arch and in a well-established interocclusal relationship, this technique is comparable to full-arch impressions [14,35]. Another reason for the divergent results may be the use of different scanners for the digital impressions in the various studies [22]. Even though the dentist has the longest working time in the chairside workflow, there are a number of benefits of this workflow compared to the conventional workflow [2]. In the chairside workflow, there is a possibility for the dentist to work on his/her own [2,3,7,14], so the restoration can be provided in 1 single day [1,2,5,7,14], which also means just one application of anesthesia [2] and no need for temporary restoration [2,13,28]. If the temporary restoration can be omitted, there will be less irritation of the tooth, which means a decreasing risk of pulpal necrosis and, therefore, fewer endodontic treatments [5,11,28]. Both conventional impression taking and scanning have a wide variation of quality [2,6], but digital impressions allow the clinician to evaluate the preparation immediately, to do corrections instantly, and to take a new impression, limited to the relevant areas [5,6,21]. Due to the standardized production process, CAD/CAM restorations are more predictable, error-free, reproducible, and of high quality. This means an increase in efficiency and, thus, a reduction in cost [1,2,3,4,5,7,13]. Digital models can be extended with more information from the patient (e.g., CT, MRI, BT) and are space-saving in comparison to traditional models [1,2,3,14,20].

The limitations of this study are that the results were achieved through in vitro testing. In vivo, handling of the camera in the mouth with the patient moving and maintaining work field isolation might be more difficult, and thus more time might be needed. Moreover, the dentists’ level of experience has an impact on the time efficiency of every workflow. The milling strategies, the material selection, and the polishing after adjustment for the chairside workflow could increase the manufacturing time. Furthermore, only one impression material with a triple tray was tested. Other materials may have a different setting time and with other trays there would be the need for an antagonist impression and a bite registration, which takes more time. 

New optimized CAD/CAM software and faster milling procedures can make digital workflows more efficient and so the cost-benefit ratio increases. Another future perspective of the development in digital workflows is given by several studies that investigate the use of 3D printing in digital workflows [36,37,38,39]. Today, conventional workflows in collaboration with dental laboratory or digital workflows with milling machines are still the preferable workflow process [36,37]. However, if 3D printing processes could increase in accuracy, aesthetic properties and time efficiency, it could be a more sustainable alternative than the milling process and the conventional methods since less waste is produced [36,37,39].

## 5. Conclusions

In general, time efficiency must be seen in combination with other advantages and drawbacks related to the different processes. However, this study provides a good basis for estimating the time required for the various digital workflows in comparison to the conventional workflow in a dental office. Within the limitations of this in vitro study, it can be concluded that: The digital labside workflow and the conventional workflow require a similar amount of time. The digital chairside workflow is more time consuming than the conventional and digital labside workflow. Within the chairside workflow, milling time is the major time-consuming factor. 

## Figures and Tables

**Table 1:** Details for all used materials with name and brand.

	Material	Name	Brand
**a**	flowable composit	Filtek Supreme XTE	3M ESPE, St. Paul, Minnesota, USA
**b**	PMMA	DD Bio-splint	Dental Direkt GmbH, Spenge, Germany
**c**	light-curing plastic	Freeprint model	DETAX GmbH & Co. KG, Ettlingen, Germany
**d**	intraoral scanner	Cerec Omnicam	Dentsply Sirona, York, Pennsylvania, USA
**e**	intraoral scanner	Trios 3	3Shape A/S, Copenhagen, Denmark
**f**	software	CEREC SW 4.5	Dentsply Sirona, York, Pennsylvania, USA
**g**	software	Trios Design Studio 1.17.2.4 & Sum 3D Dental 6.0.0.0	3Shape A/S, Copenhagen, Denmark & CIMsystems.r.l., Cinisello Balsamo, Italy
**h**	milling machine	CEREC MCXL Premium	Dentsply Sirona, York, Pennsylvania, USA
**i**	milling machine	Roland DWX-4W	Roland DG Bene-lux nv, Geel, Belgium
**j**	light-curing one-component material	Telio CS Inlay	Ivoclar Vivadent AG, Schaan, Liechtenstein
**k**	polymerisation lamp	Bluephase Polywave	Ivoclar Viva-dent AG, Schaan, Liechtenstein
**l**	prefabricated temporary composit crowns	Protemp Crown Temporization Material	3M ESPE, St. Paul, Minnesota, USA
**m**	temporary cement	Temp-Bond	Kerr GmbH, Orange, California, USA
**n**	Feldspar-blank	Vita Mark ll I14	Vita Zahnfabrik, Bad Säckingen, Germany
**o**	chairside burs	StepBur12; Cylinder Pointed Bur12; Cylinder Bur 12EF; Cylinder Pointed Bur 12EF	Dentsply Sirona, York, Pennsylvania, USA
**p**	chairside burs	1 mm Round Cylinder; 0.6 Pointed Bur	Roland DG Bene-lux nv, Geel, Belgium
**q**	ceramic polishing cups	Optrafine	Ivoclar Vivadent AG, Schaan, Liechtenstein
**r**	polishing paste	HATHO	Rodent AG, Montlingen, Switzerland
**s**	impression tray	Triple Tray	Premier Dental, Plymouth Meeting, Pennsylvania, USA
**t**	adhesive	Univesal Adhesive	Kulzer GmbH, Hanau, Germany
**u**	low viscosity vinylsiloxanether	Identium Light	Kettenbach GmbH & Co. KG, Eschenburg, Germany
**v**	high viscosity vinylsiloxanether	Identium Heavy	Kettenbach GmbH & Co. KG, Eschenburg, Germany

**Table 2:** Single steps of each workflow. The time for each step was measured using a stop clock.

	Labside	Chairside	Chairside Working Time	Conventional
Groups	CER_L/TN_L/TI_L	CER_C/TN_C/TI_C	CER_CW/TN_CW/TI_CW	CO
**Impression**	patient administration	patient administration	patient administration	impression-tray try-in
	scan	scan	scan	tray adhesive
	model calculation	model calculation	model calculation	impression taking
**CAD**		design	design	
**CAM**		preparation for milling	preparation for milling	
		milling		
**Adjustment of the Restoration**		remove sprue	remove sprue	
		approximal adjustment	approximal adjustment	
		occlusal adjustment	occlusal adjustment	
		polishing	polishing	
**Temporary** **Restoration**	fabrication of a temporary			fabrication of a temporary
	adjustment			adjustment
	remove temporarily			remove temporarily
	cleaning the die			cleaning the die

**Table 3:** Total workflow duration for each test group displayed with median and IQR.

	System	Median (Min: Sec)	IQR (Min: Sec)
**Labside**	CER_L	09:27	01:54
	TN_L	12:07	03:02
	TI_L	12:41	02:31
**Chairside**	CER_C	47:00	02:50
	TN_C	55:22	05:12
	TI_C	60:38	05:24
**Chairside Working** **Time**	CER_CW	18:32	03:13
	TN_CW	21:36	05:01
	TI_CW	25:40	05:51
**Conventional**	CO	10:39	02:24
